# Phenolics and Sesquiterpene Lactones Profile of Red and Green Lettuce: Combined Effect of Cultivar, Microbiological Fertiliser, and Season

**DOI:** 10.3390/plants12142616

**Published:** 2023-07-11

**Authors:** Milica Stojanović, Slađana Savić, Abigaël Delcourt, Jean-Louis Hilbert, Philippe Hance, Jelena Dragišić Maksimović, Vuk Maksimović

**Affiliations:** 1 Institute for Multidisciplinary Research, University of Belgrade, Kneza Višeslava 1, 11030 Belgrade, Serbia; draxy@imsi.bg.ac.rs (J.D.M.); maxivuk@imsi.bg.ac.rs (V.M.); 2 Institute for Vegetable Crops, Karađorđeva 71, 11420 Smederevska Palanka, Serbia; ssavic@institut-palanka.rs; 3 ICV-Institut Charles Viollette, UMRT 1158 BioEcoAgro, Univ. Lille, INRAE, Univ. Liège, Univ. Picardie Jules-Verne, YNCREA, Univ. Artois, Univ. Littoral Côte d’Opale, F-59000 Lille, France; abigael.delcourt@univ-lille.fr (A.D.); jean-louis.hilbert@univ-lille1.fr (J.-L.H.); philippe.hance@univ-lille.fr (P.H.); 4 Joint Laboratory University of Lille-Florimond-Desprez CHIC41Health, F-59655 Villeuneve d’Ascq, France

**Keywords:** *Lactuca sativa*, biofertilisers, phenolic acids, chicoric acid, flavonoids, luteolin-7-glucoside, sesquiterpene lactones, lactucopicrin, overall taste, UPLC/DAD/MS

## Abstract

The main goal of our study was to find an optimal combination of tested factors to achieve lettuce rich in bioactive compounds sustaining its pleasant taste. We examined three red and three green cultivars in a greenhouse using two microbiological fertilisers (EM Aktiv and Vital Tricho), and their combination. Plants were grown in three consecutive growing seasons (autumn, winter, and spring). Lactones accumulated in autumn, whereas phenolics’ concentration rose during winter. Red cultivars showed higher phenolics and lactone content, where chicoric acid and luteolin-7-glucoside were the most abundant in the ‘Gaugin’ winter trial. Lactucopicrin was the predominant lactone among tested cultivars with the highest value in the red cultivar ‘Carmesi’. Solely applicated, the fertiliser EM Aktiv and Vital Tricho led to significantly higher phenolic acid and dihydrolactucopicrin content, while combined, there were notably increased levels of all detected lactones. Application of single fertilisers had no effect on flavonoid content, while the combination even reduced it. A sensory analysis showed a negative correlation between overall taste and total sesquiterpene lactones, lactucopicrin, caffeoylmalic, and chlorogenic acid, indicating a less bitter taste with decreasing content of these compounds. Our findings indicate that the cultivar, fertiliser, and growing season jointly affected all of the tested parameters, highlighting the differences in the application of EM Aktiv, Vital Tricho, and their combination.

## 1. Introduction

Lettuce is an annual leafy vegetable crop from the Asteraceae family. Its different types have moderate requirements for climatic conditions and generally is recognized as a cool-season crop. It can be grown in different production systems (open field, covered or soilless culture) throughout a whole year, with multiple growing cycles. Covered systems provide an opportunity to cultivate lettuce during the winter in a moderate continental climate without using additional heating. Especially, with covering plants with an agrotextile, temperatures can increase up to 10 °C compared to an open field [1].

Polyphenols are a group of secondary metabolites that contribute to the protection mechanisms of plants against various abiotic and biotic stress factors [2]. As one of the main sources of dietary antioxidants, they have an important role in nutrition, with high health-benefit potential, acting as antioxidant, anti-inflammatory, and anticancer agents [3,4]. Two dominant classes of polyphenols present in lettuce are phenolic acids and flavonoids [5]. Phenolic acids are rarely found in the free form, yet in the bound form, they are mostly found as glycosides or esters. The main phenolic compounds in red leaf lettuce are caffeic acid and its derivatives (chicoric, chlorogenic, and caffeoylmalic acid) [6]. Biosynthetically closely connected to caffeic acids, quercetin and its glycosides are important phenolic compounds in a lettuce leaf [7]. Quercetin, a potent antioxidant, the main representative of flavonoids, prevents lipoprotein oxidation by scavenging free radicals [8]. Accordingly, the consumption of purple lettuce as a functional food has the potential impact on a balanced diet of evading metabolic disorders in humans, due to the prevention of gaining weight by reducing fat accumulation and simultaneously increasing energy consumption, where esculin and chlorogenic acid contribute the most [9].

Sesquiterpene lactones are terpenoids with a characteristic bitter taste, and their increased number up to 475 is found in Cichorieae [10]. In *Lactuca* species, they are accumulated inside the milk ducts and are an integral part of the latex. In damaged leaves or stems of flowering plants, when exposed to the air, latex dries and turns into a brown gummy structure known as the lettuce opium or lactucarium [11]. According to Chadwick et al. [12], this group of compounds show different roles in plants (ecological–allelopathy, antimicrobial, protective role against various stressors, contribute to bitter taste), as well as in humans (balanced nutrition diet and in pharmacy). Sesquiterpene lactones have the potential to be used for production of new pharmaceutical products with sedative and analgesic activity [13].

Three major sesquiterpene lactones are lactucin, 8-deoxylactucin, and lactucopicrin, which are the biggest contributors to the bitter taste in lettuce [14]. Their influence on taste depends on their concentration and low bitterness threshold [15]. Seo et al. [16] showed that the concentrations of sesquiterpene lactones and their total content differed significantly depending on the lettuce leaf colour (green or red) and leaf morphology (curly or cut). Among consumers, a bitter taste can be a limiting factor in acceptance and consumption of certain radicchio cultivars [17].

Taste is an important trait of interest for breeders, producers, and consumers [16,18,19]. Lettuce taste depends on the combination of the sugars, organic acids, and phenolic compounds, as well as the presence of sesquiterpene lactones, where organic acids, phenolic compounds, and sesquiterpene lactones contribute to the bitter taste, and glucose, fructose, sucrose, as well as fibres contribute to the sweet taste [16,20,21,22].

Effective microorganisms are recognized as a part of sustainable agriculture strategy. They can be used to reduce the use of mineral fertilisers and synthetic pesticides, in order to diminish the negative impact on the environment with an emphasis on reducing production costs in accordance with the environmental sustainability concept. Fertilisers with beneficial microorganisms include a mixture of different microbial strains that can be found in natural areas—photosynthetic and lactic bacteria, actinomycetes, yeasts, and fungi [23]. In applied agriculture, they can be used as inoculants, to increase the diversity and number of microflora in soil, accelerate decomposition of organic matter, stimulate plant growth and yield through enhanced nutrient availability, reduce effects of a monoculture, protect plants from pathogens, and help remove the effects of physiological disorders in plants [24,25].

*Trichoderma* spp. is one of the most important fungus genera that can be used as a biofertiliser, biopesticide, and soil improver. These are avirulent symbionts of plants—cosmopolitan fungi that can colonize roots, stimulate root growth, and improve plant nutrition, production, and resistance to various stress factors. Also, *Trichoderma* spp. can produce several secondary metabolites that can positively influence plant growth and protection from pathogens.

For research purposes, we chose six cultivars belonging to three different types (oak leaf, lollo, and butterhead Salanova), which, according to the literature, are the richest in health-benefit nutrients [5]. Moreover, previous studies indicated a higher fresh weight in green lettuce cultivars, whereas red-coloured cultivars showed higher nutritional values [26,27,28,29]. Apart from the fact that the presence and composition of bioactive compounds differ among lettuce types [5], seasonality plays a great role in defining the quality parameters of leafy lettuce [30]. The temperature and light can affect phenolic compounds especially in terms of environmental stress factors, such as chilling, a high temperature, and a high light intensity [31]. Different studies have shown the positive impact of microbiological fertilisers on the lettuce qualitative properties [32,33,34,35]. On the other hand, nitrogen fertilisation can affect lactone content [36], which could deteriorate its pleasant taste. To our knowledge, there is scarce experiments investigating and determining the effect of different fertilisation treatments combined with the cultivar and season on lettuce lactone content, especially in non-controlled production systems.

The aim of this study was to investigate the effect of the cultivar, microbiological fertiliser, and season on the content and diversity of lettuce phenolics and lactones, as well as their contribution to sensory properties. Also, this study broadens the context benefits of vegetable consumption concerning the uprising relevance of sustainable agriculture. It is expected that this study will provide a better understanding of the individual and combined effects on the phytochemical composition (specialised metabolism) of lettuce, allowing for the shaping of its food qualities (modulating the balance between health-nutrition and organoleptic properties), and ultimately formulating recommendations. With comprehensive knowledge of the interaction between plants, microorganisms, and environmental factors, it is expected to expand the practical use of biofertilisers in terms of improving the nutrient status of lettuce. Moreover, this can present an additional agricultural measure to meet the requirements for sustainable agriculture.

## 2. Results

### 2.1. Phenolic Acids

The concentration of phenolic acids in red and green lettuce was mainly influenced by the cultivar and growing season while a considerable influence of microbiological fertilisers was found in chlorogenic and caffeoylmalic acid only, as well as total phenolics (Table 1). Among this specialised metabolite class, the most abundant phenolic acid was caffeic acid, esterified by different organic acids. According to our results, two predominant phenolic acids were chicoric (di-caffeoyltartaric acid) and caffeoyltartaric acid. The red cultivar ‘Gaugin’ showed the highest level of caffeoylmalic (0.73 mg/g DW), neochlorogenic (caffeoylquinic acid) (2.89 mg/g DW), and chicoric acid (24.89 mg/g DW), while the red cultivar ‘Carmesi’ showed the highest level of chlorogenic acid (5.11 mg/g DW) and total phenolics (331.24 µg GA eq/g FW). However, the green cultivar ‘Kiribati’ showed the highest level of caffeoyltartaric acid (4.36 mg/g DW). Ferulic acid was detected in small quantities (data not shown) in specific combinations: cultivars ‘Kiribati’ (spring in all treatments), ‘Aleppo’ (autumn in all treatments), ‘Gaugin’ (autumn VT and EMA + VT, and winter C and EMA), and ‘Carmesi’ (winter in all treatments). In a similar manner, *p*-coumaric acid was detected only in the cultivar ‘Carmesi’ during the winter trial in all treatments (data not shown). Generally, red cultivars within the same type showed higher phenolic acids’ content than the green counterparts, with the exception of caffeoyltartaric acid.

Compared to the control, application of microbiological fertilisers led to an increased level of chlorogenic acid by 32.7% using the fertiliser EMA, and its application also increased the level of caffeoylmalic acid by 29.2%. The latter was increased with the application of VT by 75.0%. All applied fertilisers did not show a statistically significant contribution to the total phenolic content compared to control conditions, but among those tested, combined fertilisers led to the highest value of the total phenolic content.

The effect of the season on the content of phenolic acids led to an enhanced level of caffeoyltartaric acid by 51.1% and chicoric acid by 217.1% in the winter compared to the spring trial. Caffeoylmalic acid was increased by 1866.7% in autumn compared to spring, with an opposite situation for chlorogenic and neochlorogenic acid, where higher content was noticed in spring by 122.4% and 133.8% compared to autumn, respectively. Total phenolic content also exhibited the highest value in the autumn trial by 66.4% compared to spring. Generally, the lowest content of the majority of phenolic acids was found in the spring trial.

### 2.2. Flavonoids

The concentration of identified flavonoids was influenced by the cultivar, microbiological fertiliser, and growing season with exclusion of the fertilisers’ influence in luteolin-7-glucoside and quercetin-3-malonylglucoside-7-glucoside content (Table 2). Two major flavonoids found in tested cultivars were luteolin-7-glucoside and quercetin-3-O-(6″-O-malonyl)-glucoside. The red cultivar ‘Carmesi’ showed the highest level of quercetin-3-malonylglucoside-7-glucoside (0.85 mg/g DW), quercetin-3′-O-glucuronide (1.66 mg/g DW), quercetin-3-glucoside (1.68 mg/g DW), and quercetin-3-O-(6″-O-malonyl)-glucoside (6.37 mg/g DW), except luteolin-7-glucoside, which was predominant in the red cultivar ‘Gaugin’ (6.96 mg/g DW). Among mentioned flavonoids, lettuce samples showed the presence of rutin (quercetin-3-rutinoside) and quercetin-3-malonylglucoside-7-glucuronide too (data not shown). A rutin derivate was only present in red cultivars ‘Carmesi’ (winter in control, EMA, VT), ‘Gaugin’ (autumn and winter in control), and ‘Murai’ (winter in EMA + VT). Quercetin-3-malonylglucoside-7-glucuronide was detected in cultivars ‘Gaugin’ (autumn, winter in all treatments, and spring in EMA and VT), ‘Aleppo’ (winter in control), ‘Murai’ (winter in all treatments), and ‘Carmesi’ (autumn and winter in all treatments). For all tested flavonoids, red cultivars showed a tendency of higher accumulation compared to green cultivars.

Application of combined fertilisers led to a decreased level of quercetin-3-glucoside by 21.7%, quercetin-3-O-(6″-O-malonyl)-glucoside by 22.3%, and quercetin-3′-O-glucuronide by 25.6%, while there was no effect on the two remaining compounds.

The winter experiment was depicted by the highest level of all flavonoids characterized by a markedly elevated concentration of quercetin-3-glucoside by 320.0%, quercetin-3-O-(6″-O-malonyl)-glucoside by 448.8%, and quercetin-3′-O-glucuronide by 235.4% compared to the spring trial. Luteolin-7-glucoside content was increased by 263.7% in winter compared to the autumn experiment. The highest variation in the values was spotted for quercetin-3-malonylglucoside-7-glucoside by 2533.3% in autumn compared to spring.

### 2.3. Sesquiterpene Lactones (STL)

The concentration of total sesquiterpene lactones, lactucopicrin, and dihydrolactucopicrin was also influenced by the cultivar, microbiological fertiliser, and growing season (Table 3). The red cultivar ‘Carmesi’ showed the highest value of total sesquiterpene lactones (0.39 mg/g DW) and lactucopicrin (0.37 mg/g DW), whereas the highest level of dihydrolactucopicrin was found in the green cultivar ‘Aquino’ (0.014 mg/g DW). As presented in Table 3, the predominant sesquiterpene lactone in tested cultivars is lactucopicrin. Simple lactones, such as lactucin and dihydrolactucin, were not detected in all samples (data not shown). Lactucin was generally present in cultivars ‘Kiribati’ (autumn, with treatments C, EMA, VT) and ‘Carmesi’ (in winter with VT and EMA + VT, and in spring with EMA and EMA + VT). Dihydrolactucin was found only in two samples of the cultivar ‘Aquino’ (in autumn with EMA + VT, and in winter using the fertiliser EMA).

**Figure 1:**
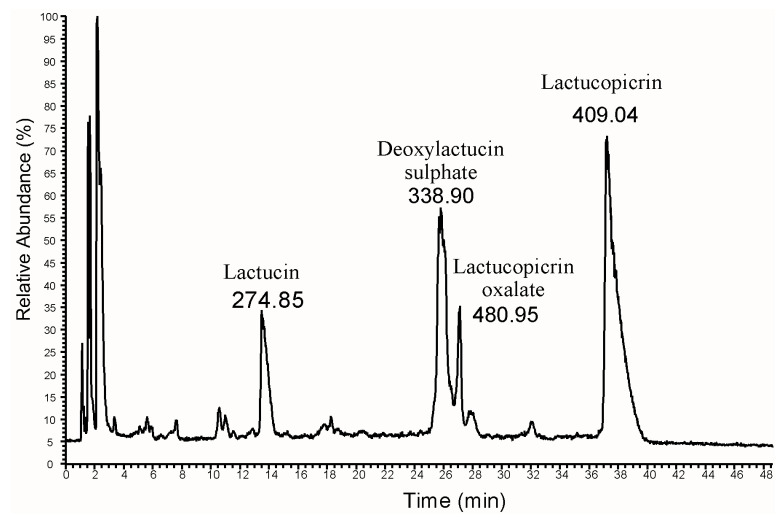
HPLC MS base peak chromatogram of detected sesquiterpene lactones in the lettuce latex.

A preliminary HPLC MS analysis of latex collected from all six cultivars that was performed for the autumn trial showed the presence of lactucin, deoxylactucin sulphate, lactucopicrin, and lactucopicrin oxalate (Figure 1). This analysis additionally denoted a derivate of lactucopicrin as a predominant form of sesquiterpene lactones.

In general, the application of microbiological fertilisers increased the concentration of sesquiterpene lactones in all cultivars. The combination of both fertilisers (EMA + VT) led to an increased concentration of STL by 66.7% and lactucopicrin by 40.0%. Compared to the control, all fertilisers increased the concentration of dihydrolactucopicrin in the range 133.3–200.0%, especially VT, leading to the highest increase detected.

Concentrations of STL and lactucopicrin were significantly higher (100.0%) in the autumn experiment compared to winter. On the contrary, the highest content of dihydrolactucopicrin was found in spring, and it was 266.7% higher than in the winter experiment.

### 2.4. Sensory Analysis

Remarks for the overall taste ranged from 1–5, where a score of 1 referred to a very poor overall taste, while a score of 5 referred to a very good overall taste in lettuce. According to sensory evaluation panel findings, all cultivars showed a score between 2 and 3, indicating that all cultivars had a poor (2) to neutral–acceptable taste (3). A very poor and poor taste was associated with a bitter taste, which aggravated the overall taste and final perception of the sample. Results of the sensory analysis showed a significant influence of the season on the lettuce overall taste, excluding the effect of the cultivar and microbiological fertiliser (Table 3). The most favourable overall taste was obtained in the winter experiment, leading to an increased remark of the taste by 16.5% compared to autumn.

### 2.5. Correlations

Results of all tested phenolic and lactone compounds, their total content, and overall taste were used to determine possible correlations (Table 4). Chicoric acid and luteolin-7-glucoside, as predominant phenolics, exhibited similar linear positive correlations with other phenolics, yet the only one negative correlation detected was with dihydrolactucopicrin, pointing out that with an increase of phenolics, the content of dihydrolactucopicrin is reduced.

A very strong positive correlation was found between chicoric acid and luteolin-7-glucoside (r = 0.97 **) and quercetin-3-O-(6″-O-malonyl)-glucoside (r = 0.86 **); a strong positive correlation was found with quercetin-3-glucoside (r = 0.72 **)**,** quercetin-3′-O-glucuronide (r = 0.71 **), and quercetin-3-malonylglucoside-7-glucoside (r = 0.67 **); a moderate positive correlation was found with neochlorogenic acid (r = 0.56 **), TPC (r = 0.55 **), and caffeoyltartaric acid (r = 0.41 **); a weak positive correlation was found with caffeoylmalic acid (r = 0.29 **); and a very weak positive correlation was found with the overall taste (r = 0.15 *). In contrast, a negative weak correlation was found with dihydrolactucopicrin (r = -0.24 **).

Luteolin-7-glucoside showed a very strong positive correlation with chicoric acid (r = 0.97 **) and quercetin-3-O-(6″-O-malonyl)-glucoside (r = 0.82 **); a strong positive correlation with quercetin-3-glucoside (r = 0.66 **)**,** quercetin-3′-O-glucuronide (r = 0.66 **), neochlorogenic acid (r = 0.65 **), and quercetin-3-malonylglucoside-7-glucoside (r = 0.61 **); a moderate positive correlation with TPC (r = 0.54 **); a weak positive correlation with caffeoyltartaric acid (r = 0.29 **) and caffeoylmalic acid (r = 0.25 **); and a very weak positive correlation with the overall taste (r = 0.16 *). In contrast, a negative weak correlation was found with dihydrolactucopicrin (r = −0.24 **).

Lactucopicrin, as a predominant lactone, showed a very strong positive correlation with STL (r = 0.96 **), a weak positive correlation with TPC (r = 0.27 *) and dihydrolactucopicrin (r = 0.26 **), and a very weak positive correlation with chlorogenic acid (r = 0.19 **) and quercetin-3-malonylglucoside-7-glucoside (r = 0.17 *). In contrast, a very weak negative correlation was exhibited with the overall taste (r = −0.16 *) and caffeoyltartaric acid (r = −0.15 **).

The overall taste attribute showed a very weak positive correlation with luteolin-7-glucoside (r = 0.16 *), chicoric acid (r = 0.15 *), and neochlorogenic acid (r = 0.15 *). As an opposite, a very weak negative correlation was found with STL (r = −0.16 *), lactucopicrin (r = −0.16 *), chlorogenic (r = −0.16 *) and caffeoylmalic acid (r = −0.14 *). These results indicated deterioration of the overall taste with the increasing content of chlorogenic and caffeoylmalic acid, lactucopicrin, and STL.

## 3. Discussion

Total phenolic content in lettuce depends on the influence of the cultivar, physiological, and agroecological conditions [26]. Generally, our results pointed out that red cultivars had higher TPC compared to their green counterparts, which is in agreement with previous reports [26,28,37,38]. A possible reason for the lower TPC of green cultivars could be due to the lower content, or even absence, of anthocyanins [5]. Among all tested cultivars, the red cultivar ‘Carmesi’ had the highest total phenolic content (Table 1). Similarly to our research, Sytar et al. [39] pointed out the same cultivar as the most abundant in TPC, compared to other red and green lettuce cultivars, almost in all growing conditions. Also, our TPC content is in line with previously reported values by Oh et al. [31], who reported TPC of 0.1–0.35 mg GAE/g FW, and Gan and Azrina [40], who reported values of 4.85–76.05 mg GAE/100 g FW.

The most abundant phenolic acids detected in our experiments were chicoric, caffeoyltartaric, and chlorogenic acid (Table 1). In accordance with our findings, many studies showed that chicoric acid was predominant compared to other phenolic compounds in lettuce [41,42,43]. Levels of chicoric acid that we detected in six cultivars (Table 1) are in range with the results of Nicole et al. [44], in which the content in lettuce ranged from 5.58–11.20 mg/g DW. Furthermore, our red pigmented cultivars exhibited an even higher amount of chicoric acid than in the research of Assefa et al. [45], who reported values of 337.1–19,957.2 µg/g DW. Ribas-Agustí et al. [46] found that *p*-coumaric acid derivatives were sporadically detected, but below the level of quantification. Similarly, in our trials, *p*-coumaric acid was not detected in all samples.

Main flavonoids in our samples were luteolin-7-glucoside and various quercetin derivates (Table 2). Green and red Salanova lettuce exhibited a higher content of luteolin-7-glucoside, quercetin-3-glucoside, and quercetin-3′-O-glucuronide than the same variety in the research of Giordano et al. [47]. Even though, results of some previous research for the quercetin derivates are in accordance with our samples [6,43].

Above-mentioned results stand in agreement that the cultivar had a great impact on the polyphenol synthesis, accumulation, and profile, while individual content is dependent on the environmental factors [28,48,49]. As previously reported, there is a difference in the profile composition of polyphenols between lettuce types and cultivars (green and red) [28], showing that caffeic acid derivatives were the main polyphenols in green cultivars, while flavonols were observed in larger quantities in red cultivars, having in mind that anthocyanins can be found only in red leaf lettuce. Our results support such findings, where red cultivars ‘Carmesi’ and ‘Gaugin’ were characterized as the most abundant with phenolic acids and flavonoids, with an exception of a high content of caffeoyltartaric acid in the green cultivar ‘Kiribati’. Research by Ferreres et al. [50] showed that Lollo Rosso is much richer in phenolic acid derivatives than other previously studied cultivars. An explanation for the higher content of phenolics in red compared to green lettuce cultivars could be due to a different allocation of resources, which in red cultivars is probably connected with an enhanced synthesis of phenolic compounds rather than yield [51].

In our samples, phenolic acids were more prominent than flavonoids, similar to the work of Nicolle et al. [44], where their low amounts may be due to the apparent cultivar or the ability to synthesize flavonoids in lettuce leaves. Different concentrations of phenolic acids that were found in lettuce samples can be linked to different growing conditions, since the environment significantly impacts phenolic composition [44]. Even though phenolic acids were more dominant in lettuce samples, chicoric acid showed a very strong positive correlation with luteolin-7-glucoside and various quercetin derivates, meaning that enhancement of this phenolic acid increases the content of a quercetin derivate. A similar positive trend in correlations between chicoric acid and two quercetin derivates was observed in the study of Assefa et al. [45]. Since this positive trend was observed, it is important to discover different methods to enhance the content of polyphenols in lettuce, as they have an important protective role in the human diet. As polyphenols, phenolic acids are powerful antioxidants and have been found to exhibit antibacterial, antiviral, anticancer, anti-inflammatory, and vasodilator effects [5]. The biological effect of caffeic acid derivatives is reflected, among others, in the inhibition of LDL cholesterol oxidation [52], as well as in both the promotion of apoptosis and inhibition of tumour cell growth [53]. Moreover, luteolin and chicoric acid synergistically inhibited inflammatory activity [54]. So, the first step towards producing lettuce with “additional bioactive value” is to select the optimal cultivar, and to choose an adequate lettuce type in production.

Regarding the application of microbiological fertilisers and their influence on the total and individual phenolic content, there is a limited number of studies with inconsistent results. In our experiments, application of all fertilisers did not significantly influence TPC in comparison to control conditions (Table 1). Between treatments, application of combined fertilisers led to a higher content of TPC compared to the single fertiliser EMA. Positive examples of application of biofertiliser with an increased content of total phenols were reported in lettuce leaves based on a yeast extract [55]. Also, the association of lettuce with mycorrhizal fungi showed a tendency to increase the content of total phenols in lettuce [56]. In contrast, the association between *Salvia officinalis* and *Glomus intraradices* reduced TPC in the *Salvia* leaves [57].

In our research, the most abundant phenolic acids (chicoric and caffeoyltartaric acid) were not affected by fertilisation treatments (Table 1). In contrast, there was a positive effect of the fertilisers EMA and VT on chlorogenic and caffeoylmalic acid in the range 29.2–75.0%, while application of combined fertilisers led to a decreased level of three quercetin derivates in the range 21.7–25.6%. Literature data showed that the content of the main polyphenolic compounds in the red cultivar ‘Tuska’ was less affected by the type of fertiliser used, while the polyphenols in green lettuce cultivars (Batavia ‘Maritima’ and ‘Winter Butterhead’) were significantly changed by the use of the organic fertiliser Arkobaleno and biofertiliser EKOprop NX [58]. The same authors indicated that the use of mineral, organic, and biofertilisers had a minor effect on the polyphenol content. The research of Ayuso-Calles et al. [59] showed that after inoculation with *Rhizobium*, all detected phenolic acids were significantly higher, including caffeoyl derivatives (dicaffeoylquinic and chicoric acid), as well as flavonoids compared to non-inoculated plants—control conditions. Above-mentioned comparisons with our findings indicate a need of further investigation with a different concentration of applied fertilisers during cultivation in prolonged studies and seasons.

In contrast to the selected fertiliser, the season had a significant impact on all phenolic acids and flavonoid levels, as well as in the interaction with the cultivar (Table 1 and Table 2). A great number of studies were conducted under controlled environmental conditions, which inherently discard an impact and interactions of disturbing factors existing in an open field or in a protected environment without additional heating and lighting. Marin et al. [60] found that climatic factors influenced the phenolic content and particularly enhanced the temperature and radiation during the season, leading to an increased content of phenolic acids and flavonoids. Apart from those mentioned, various abiotic and biotic stress factors and the application of agricultural practices can have an important influence on the phenolic levels and profile in lettuce [61].

Climatic data from our experiments pointed out that average day/night temperatures in autumn (11.9/5.7 °C) and winter (10.8/1.8 °C) were suboptimal, comparing to the lettuce optimal ambient temperature demands. During the winter trial, 7 days after transplanting, plants were exposed to cold stress that lasted for 56 days, in which daily temperatures were below 0 °C. Average day/night temperatures in spring were almost optimal (26.3/15.3 °C), with the remark that during the day, plants in a greenhouse were exposed to temperatures above 30 °C.

Quantitatively predominant phenolic acids (chicoric and caffeoyltartaric acid) and the most flavonoids were accumulated during the winter trial, which can be explained in a way that cold stress at the beginning of the experiment and suboptimal temperatures during the vegetation period led to a higher content of these compounds. The activity of phenylalanine ammonia-lyase (PAL), which catalyses the conversion of phenylalanine to cinnamic acid, was higher in lettuce grown at 13/10 °C and 20/13 °C compared to higher day/night temperature regimes, such as 25/20 °C and 30/25 °C [62]. These results indicated that lower temperatures during the photoperiod enhanced PAL activity, resulting in intensive cinnamic acid, and consequently, overall phenolic acid biosynthesis.

In contrast, in our trials, chlorogenic and neochlorogenic acid accumulated in spring, with higher temperatures and day light compared to the autumn and winter trial. Similar to our research, the investigation of Sublett et al. [63] showed a significant effect of the season, as well as the interaction between the cultivar and season, on phenolic content in lettuce grown in a greenhouse, where the chlorogenic acid content was the highest in spring, being 73% higher when compared to autumn. Liu et al. [26] noticed that lettuce harvested at both higher temperatures and light intensities had higher phenolic contents (by 6.1%) compared to plants harvested under relatively lower temperature and light intensity conditions. Our results for chlorogenic and neochlorogenic acid are consistent with research indicating that a higher radiation in spring increased phenolic content possibly as a higher stimulation of phenylpropanoid pathway gene expression [60,64].

Furthermore, protected environment cultivation (such as high tunnel or greenhouse) tends to lower the content of phytonutrients compared to open field cultivation [65], mainly in a way that materials for covering greenhouses and high tunnels decrease light quality and intensity. Lettuce grown in a polycarbonate greenhouse had a lower flavonoid content than in an open field [48]. The extent to which high tunnels and greenhouses can diminish phenolic content is still unknown. Zhao et al. [61] investigated the content of phenolic compounds in green and red lettuce grown in high tunnels, showing its reduction in both green and red lettuce, though it was more pronounced in the latter. Our results from the spring trial showed the decreased content of luteolin-7-glucoside was within the range obtained by previously mentioned authors, even for plant samples from an open field, while in the case of chlorogenic acid, higher values were achieved in our experiment compared to tunnels and open field experiments. Oh et al. [66] found that the polyethylene film covering the high tunnel transmitted only 50–60% of the light, which surely had a negative role in photosynthesis as well as the production of secondary metabolites. Polyethylene film was in the second year of use in our trials (optimal period according to the manufacturer’s recommendation) so it can be assumed that it had no negative impact on the phenolic content in terms of reduced or changed quality and transmission of light. The mentioned inconsistency in results suggests the complex interconnection of secondary metabolism and antioxidative protection as an integral part of the lettuce adaptation mechanism regarding growing conditions.

According to the presented data, the cultivar and season jointly had a significant effect on all phenolic acids and flavonoid content. Microbiological fertilisers did not affect dominant phenolic acids (chicoric and caffeoyltartaric acid) and a flavonoid (luteolin-7-glucoside), which could lead to the simple decision not to apply these fertilisers in production. For particular phenolic acids—chlorogenic and caffeoylmalic—these fertilisers led to an increased level of these two acids. Furthermore, for phenolic acids that were not affected, treatments had higher values than control plants. This needs further investigation, with a possible enhancement of the fertiliser dosage during different seasons and their long-term application. Nevertheless, our experiments showed that the interaction between all investigated factors gave a statistically significant impact on the measured parameters, indicating a necessity for statistical evaluation of their combined effect presented in Table 1 and Table 2.

The literature data showed that the three most abundant sesquiterpene lactones in wild lettuce are lactucin, 8-deoxylactucin, and lactucopicrin [18]. Our trials with six cultivars pointed out that lactucopicrin stands as the dominant sesquiterpene lactone in leaf samples, unlike lactucin and dihydrolactucin, additionally being confirmed by a very strong positive correlation between contents of lactucopicrin and total lactones (Table 3 and Table 4). Previous research on chicory also depicted lactucopicrin as the major lactone, contributing to a bitter taste, as a result of both its concentration and lower bitterness threshold [67]. In our experiments, lactucin and dihydrolactucin were not found in all samples, which can be related to genetic variation and environmental conditions.

Bitter compounds are differently distributed within the plant, with higher concentrations found in the white latex, while significantly lower STL concentrations are found in iceberg lettuce leaves when compared to roots [68]. Before we performed lactone quantification from leaves, a pilot experiment was conducted in order to determine their qualitative presence in the latex (Figure 1). A different qualitative lactone profile was obtained from the latex compared to leaf samples. Again, lactucopicrin was found as a major compound in both leaf and latex samples.

The present study showed a significant influence of the cultivar on all tested lactones, which is in accordance with a previously described important impact of the cultivar on sesquiterpene lactone content [14]. Leafy cultivars (var. *crispa*) in our experiments showed higher lactone values compared to the head type (var. *capitata*). Literature data confirmed that leaf lettuce commonly has a more bitter taste than other types (head lettuce, romaine), while the bitter taste of chicory and endive was two to three times higher than in lettuce [14]. Furthermore, red cultivars in our trials showed a tendency for an enhanced content of lactucopicrin and total lactones compared to the green counterparts. Similarly, the research of Seo et al. [16] showed that the concentrations of individual and total sesquiterpene lactones differed significantly depending on the lettuce leaf colour (green or red) and leaf morphology (curly or cut), gaining higher values in red lettuce with curly leaves. The cultivar ‘Carmesi’, as a red curly cultivar, accumulated the highest content of lactucopicrin and total lactones (Table 3). Results of average concentrations of different lactones found in our samples are lower than in the study of Sung et al. [19]. This discrepancy in concentration can be explained by the fact that lactones are functional compounds with a tendency to change their concentration during plant growth and development, as well as a contribution of different cultivar and environmental conditions in different experiments.

Application of microbiological fertilisers had a significant influence on individual and total lactone content, emphasizing the effect of combined fertilisers (Table 3). According to our study, combined fertilisers led to an increased level of lactucopicrin and STL by 40.0% and 66.7%, respectively, while all applied fertilisers led to an increased level of dihydrolactucopicrin in the range 133.3–200.0%. This is an important fact for lettuce producers, since lactones contribute to a bitter taste, which can be a limiting factor in different cultivar acceptance on the market. Literature data are lacking in results of experiments with microbiological or other types of fertilisers on lactone content and a change in their concentration. Some rare research showed that the application and dosage of a nitrogen fertiliser can significantly affect the concentrations of lactucin and lactucopicrin [36].

Besides the cultivar, environmental factors, date of planting, and post-harvest factors (harvest time, temperature) also influence lactone content. Our results showed the great impact of seasonality on lactone content (Table 3). Lactucopicrin and total lactones were highly accumulated in autumn, but dihydrolactucopicrin was highly accumulated in the spring trial. Air temperatures in the autumn trial were more suboptimal with an additional effect of a short day, comparing to the spring trial. Our findings for lactucopicrin and total lactones found in leaf samples are not in accordance with the statement that the concentration of bitter compounds in lettuce increases with the plant developmental stage and with an increasing air temperature [69]. Furthermore, there is a stronger appearance of sesquiterpene lactones in the lettuce latex, especially in the flowering phase and shortly before flowering [70]. Since, in all trials, plants were harvested before flowering with no signs of bolting noticed, detected lower amounts of lactones can be explained from this aspect. Apart from the missing literature data for fertilisation treatments, there is also scarce evidence of different trials regarding a seasonal impact on the lactone content and profile, which could explain the impact of environmental factors on lactone content. Our results indicated not only the significant influence of a single factor but also that multifactor interactions were statistically significant, requiring a careful approach in the selection of cultivars and fertilisers in relation to the season.

According to the sensory attribute tests, overall taste was scored towards around neutral–acceptable (2.83–3.08) for almost all cultivars, which could be interpreted in a way that lactones and phenolics did not aggravate lettuce taste. A very weak negative correlation between lactucopicrin, STL, chlorogenic and caffeoylmalic acid, and overall taste could indicate that these compounds could have an impact in consumer acceptance of different cultivars (Table 4). Similar results with the contribution of lactucopicrin to the perception of bitterness were noticed in endive and escarole samples [71]. The same authors found out that a bitterness perception could be affected by the balance between compounds influencing in the same direction. Consequently, the reduction in a different STL content in food, through plant breeding or processing, is an uprising trend in agricultural production [72]. Differences in the profile of sesquiterpene lactones in cultivated and wild *Lactuca* species, where interspecies hybrids were designed with crossing, indicated that it is possible to identify the genes responsible for the composition of sesquiterpene lactones and thus create lines characterized by the absence or presence of certain lactones that are responsible for the bitter taste [70]. Additionally, experiments with different coffee extraction methods pointed out a positive correlation between the concentration of chlorogenic acid and perception of bitterness and astringency [73].

From all three investigated factors, only the season showed a significant single impact on overall taste (Table 3). The most favourable taste was found in the winter/spring experiment, while the lowest score was found in autumn. Lactucopicrin accumulated in the autumn trial, which can be the cause of the least pleasant taste. Furthermore, the interaction between the cultivar, fertiliser, and season was statistically significant, leading to the conclusion of a combined effect not only on overall taste but also on phenolics and lactone content. This implies required additional research on aspects of cultivars, fertilisation, and seasonality in order to obtain lettuce rich in bioactive compounds with a pleasant taste.

## 4. Materials and Methods

### 4.1. Plant Material

Lettuce experiments included six Rijk Zwaan cultivars with green- and red-coloured leaves. All genotypes are associated with three lettuce types: oak (green ‘Kiribati’, red ‘Murai’-L. *sativa* var. *crispa*), multi-leaf butterhead Salanova^®^ (De Lier, the Netherlands) (green ‘Aquino’, red ‘Gaugin’-L. *sativa* var. *capitata*), and lollo (green ‘Aleppo’, red ‘Carmesi’-L. *sativa* var. *crispa*). Lettuce seedlings were grown in peat cubes (Potgrond H, Klasmann-Deilmann GmbH, Geeste, Germany) that were mechanically made to be a size of 4 cm in a glasshouse of the company Grow Rasad, Irig, Serbia. Seedling production endured for 20 days in autumn, 39 days in winter, and 21 days in spring. Before transplanting into a greenhouse, seedlings were acclimatised for a couple of days in the greenhouse. All cultivars were transplanted in the phase of the fourth true leaf unfolding.

### 4.2. Microbiological Fertilisers

Two different microbiological fertilisers and their combination were applied on lettuce plants. EM Aktiv (EMA, Candor, EM tehnologija Ltd., Valpovo, Croatia) is a liquid medium that contains plant extracts derived from the mixture of organic matter performed with microbiological fermentation of molasses from sugar cane. Vital Tricho (VT, Candor, EM tehnologija Ltd., Valpovo, Croatia) is a powder combination of two species: *Trichoderma viride* and *Trichoderma asperellum* (5 × 10^9^ CFU/mL).

### 4.3. Experimental Design and Climate Data

Lettuce plants were grown at the company Iceberg Salat Centar (Surčin, Serbia) in three continuous growing seasons: (1) autumn from October to December 2016; (2) winter from December 2016 to April 2017; and (3) spring from April to June 2017. The plants were cultivated in the greenhouse without additional heating and lighting, with an area of 256 m^2^. Prior to the initial planting, a physical and chemical analysis of the soil was carried out. The physico-chemical analysis showed that the soil was black marsh and clay loam, which was sufficiently enriched with total nitrogen (0.22%), readily available phosphorus (58.35 mg/100 g), readily available potassium (32.45 mg/100 g), and humus (5.02%). The soil was used in the intensive vegetable production and no microbiological fertilisers were applied before these experiments. According to the chemical analysis of the soil, it was decided that all treatments would be carried out without using inorganic fertilisers.

The experiments were organized as a complete block design with four treatments: (I) control (C)—without applying any fertiliser; (II) the fertiliser EM Aktiv (EMA); (III) the fertiliser Vital Tricho (VT); and (IV) a combination of fertilisers EM Aktiv and Vital Tricho (EMA + VT), in 3 replications. Main plots were sized at 2 × 1 m with 32 plants in each plot, a density of 25 × 25 cm, a distance between repetitions of 50 cm in each treatment, and a distance between treatments of 100 cm.

After soil preparation, microbiological fertilisers were applied in the soil (150 mL of EMA, 21 g of VT, and 150 mL + 21 g of EMA + VT for each treatment dissolved in 10 l of water) and the soil was covered with a black mulch film. During the lettuce vegetation period, microbiological fertilisers were applied four times foliar (30 mL of EMA, 12 g of VT, and 30 mL + 12 g of EMA + VT for each treatment dissolved in 6 l of water) using a battery sprayer.

In the lettuce cultivation, regular agricultural practices were applied (hoeing, weeding, irrigation, preventive protection against pests and diseases, and ventilation). All plants were harvested by hand on the same day when they reached the commercial size and technological maturity indicated by head compactness.

Climate data were measured using a RC-4HC Data Logger (Elitech Technology Inc., San Jose, CA, USA), which collected air temperature and air humidity data for 24 h. The photoperiod was obtained using data for the location of the greenhouse, Surčin, Serbia (Weather Underground, https://www.wunderground.com (accessed on 6 June 2017)) and plants were exposed to different day lengths in all seasons (Table 5).

### 4.4. Sample Preparation for Chemical Analysis

After harvesting, fresh leaf samples were kept in plastic bags and stored in a refrigerator at −20 °C for a further analysis. Frozen samples were milled in liquid nitrogen, homogenized and extracted in 80% ethanol, and centrifuged at 10,000× *g* for 10 min, and a supernatant was used for an analysis of total phenolic content. Individual phenolic compounds were determined from frozen samples that were homogenized in methanol (80%) acidified with formic acid, 98:2 (*v*/*v*). Lyophilization was performed for 48 h, after which the freeze-dried samples were packed in hermetically sealed plastic bags and stored in a freezer at −20 °C for a further lactone analysis.

To quantify all STL (hydroxyl, oxalyl, and glycosyl groups), extraction from the freeze-dried and ground leaves included maceration over a sufficient time period to promote hydrolysis on carbon 15 of the cyclopentenone ring [74]. In total, 300 mg of the freeze-dried lettuce leaf powder was mixed with 9 mL of methanol/water/acetic acid (75:23:2, *v*/*v*/*v*) [75], vortexed for a few seconds, and shaken at 1000× *g* (Thermomixer, Eppendorf, Montesson, France) for 17 h at 25 °C. The extract was centrifuged at 15,000× *g* (Sigma Centrifuge, Sigma Laboratory Centrifuges, Osterode, Germany) at 4 °C for 10 min and a supernatant (7 mL) was evaporated under reduced pressure with a vacuum concentrator. The dried extract was resuspended with 4 mL of water and vigorously vortexed. The aqueous phase was partitioned with 4 mL of ethyl acetate to remove sugars [17] and the organic phase was separated with centrifugation. The partitioning step was repeated twice and ethyl acetate fractions were pooled and evaporated under reduced pressure. Finally, the dried extract was dissolved in 80% methanol and used for a further analysis.

Sesquiterpene lactones from the white latex were analysed in the initial autumn trial using small V cuts on the core of lettuce rosette in all six cultivars. Latex drops were collected and 10 µL of the sample was mixed with 1 mL of methanol containing 1% phosphoric acid. Samples were centrifuged at 16,000× *g* for 10 min and a supernatant was filtered through a 0.45 µm membrane [70].

#### 4.4.1. Total Phenolic Content (TPC) Determination

Total phenolic content was determined using a Folin–Ciocalteu reagent according to [76]. Gallic acid was used as a standard to create a calibration curve (0–340 mg of GA/mL). Absorbance was recorded at 724 nm using a spectrophotometer (Multiskan Spectrum, Thermo Electron Corporation, Vantaa, Finland). Total phenolic content is expressed as micrograms of gallic acid equivalents per gram of the fresh weight (μg GA eq/g FW).

#### 4.4.2. Detection of Individual Phenolic Compounds

Samples were injected into a Waters HPLC system consisting of a 1525 binary pump, thermostat, and 717+ autoinjector connected to a Waters 2996 Diode Array (DAD) and an EMD 1000 quadrupole detector with an electron spray ionization (ESI) probe (Waters, Milford, MA, USA). Separation of phenolic compounds was performed using a Symmetry C-18 reversed-phase column of dimensions 125 × 4 mm packed with 5 µm diameter particles (Waters, Milford, MA, USA), connected to a pre-column. Two mobile phases, A (0.1% formic acid) and B (acetonitrile), were used at a flow rate of 1 mL/min in the following gradient profile: initial 10% B, followed by a linear increase to 50% B over the next 35 min, then 10 min of reduction to 10% B with an additional 5 min of equilibration. A post column flow splitter (ASI, Richmond, CA, USA) with a 5/1 ratio was used to obtain the optimal flow rate (0.2 mL/min) of the mobile phase for the simultaneous MS analysis. DAD chromatograms were recorded at 330 and 380 nm for the detection of phenolic acids and flavonoids, respectively. The quantification of the compounds was performed with the external standard method, using analytical standards for phenolic acids and flavonoids obtained from the Sigma-Aldrich corporation (St. Louis, MO, USA). Due to the lack of the specific standards, values for quercetin-malonylglucosides are expressed in gram equivalents of quercetin, calculated with the external standard method. Peak validation was accomplished with a LC/MS analysis, where the signals were detected in the negative scanning mode (range, 100–900 *m*/*z*) with the following ESI source parameters: capillary voltage of 3.0 kV; voltage on the cone of −35 V; and the extractor voltage and RF lens voltages were 3.0 and 0.2 V, respectively. The source temperature and desolvation temperature were 130 and 400 °C, respectively, in a N_2_ flow of 500 L/h. Data acquisition, processing, and peak confirmation were performed using Waters Empower 2 software (Waters, Milford, MA, USA). The individual phenolic compounds’ content is expressed in milligrams per gram of the dry weight (mg/g DW).

#### 4.4.3. Sesquiterpene Lactone (STL) Analysis

The analysis of the white latex was performed using an Ultimate 3000 UHPLC system connected to a triple quadrupole mass spectrometer (TSQ Quantum Access Max, Thermo Fisher Scientific, Bremen, Germany). Chromatographic conditions were the same as for the phenolic compounds’ analysis. A TSQ Quantum Access Max mass spectrometer equipped with a HESI source was used with the vaporizer temperature kept at 380 °C and the following ion source parameters: spray voltage of −3500 V, sheath gas (N_2_) pressure of 45 AU, ion sweep gas pressure of 2.5 AU and auxiliary gas (N_2_) pressure of 15 AU, capillary temperature at 350 °C, and skimmer offset of 0 V. The MS data were acquired in the negative scan mode and in the *m/z* range from 100 to 950. The Analyst version of 1.4 Xcalibur software (Thermo Fisher Scientific, Bremen, Germany) was used for data acquisition and processing.

The quantification of STL from dried methanol extracts was performed using an Ultimate 3000RS UPLC system (Thermo Fisher Scientific, Villebon-sur-Yvette, France) consisting of a quaternary pump, a column oven, and a UV-visible diode array detector. A Kinetex PFP column (100 × 4.6 mm, 2.6 𝜇m) (Phenomenex, Le Pecq, France) was used for compound separation. The elution was performed with water (A), methanol (B), and acetonitrile (C), all acidified with 0.1% phosphoric acid, using the following gradient at a flow rate of 1.1 mL/min: 0–14.5 min, 0–64.5% B; 14.5–15.5 min, 64.5–0% B; 15.5–20 min, 0% B; and 0–20 min, 4% C. The oven temperature was 45 °C and the injection volume was 5 µL. Sesquiterpene lactones were determined at 254 nm with external calibration using dihydrolactucin (3810, Extrasynthese, Genay, France), lactucin (3809, Extrasynthese, Genay, France), dihydrolactucopicrin (3811, Extrasynthese, Genay, France), and lactucopicrin (3813, Extrasynthese, Genay, France) standards. Sesquiterpene lactone content is expressed as milligrams per gram of the dry weight (mg/g DW).

#### 4.4.4. Sensory Analysis

The sensory panel was organized at the company Iceberg Salat Centar, Surčin, Serbia. Before sensory testing, context and rules were briefly explained to the panellists, as well as evaluation of overall taste. Evaluators received training according to the general guidelines (ISO 8586:2012) [77] for the selection, training, and monitoring of selected assessors and expert sensory assessors. Panellists were trained to recognize primary taste bitterness, sweetness, saltiness, and sourness. They received five solutions (the control solution for all tastes was water) and they were asked to rank samples in terms of intensity (from the weakest to the strongest intensity).

The sensory analysis was performed on the day of the harvest with 4 panellists (2 men and 2 women) consisting of agriculture faculty students. All damaged, discoloured, outer leaves were removed before washing with water and drying with paper towels. Panellists were separated and an evaluation sheet and a pencil were placed in front of each. Samples were packed in plastic bags coded with digits and provided to the panellist, after which remarks were written on the paper sheet for each sample. Whole fresh leaf samples were distributed to the evaluators and every treatment was analysed with 12 samples. Evaluators were encouraged to state as precisely as possible what they feel, in order to obtain the most detailed evaluation results. Water and toasted bread were provided for cleansing the palate between samples. Overall taste was evaluated using a 5-point hedonic scale and lettuce samples were labelled as very poor taste—score 1, poor taste—score 2, neutral–acceptable taste—score 3, good taste—score 4, and very good taste—score 5 [78].

### 4.5. Statistical Analysis

A three-way ANOVA with a Tukey’s test for post hoc comparation was used to test the effect of the cultivar, fertiliser, and season. All results were calculated at a significance level α of 0.05. Pearson’s correlation was used to test the possible correlation between observed parameters. The statistical analysis was performed using the software SPSS Statistics (Version 25.0.; Armonk, NY, USA: IBM Corp) and Microsoft Office Excel 2019 (Microsoft Corp., Redmond, WA, USA).

## 5. Conclusions

The highest content among the phenolic acids and flavonoids in lettuce was chicoric acid and luteolin-7-glucoside, respectively. Among STL, lactucopicrin was the most abundant compound and the major contributor to a disagreeable taste. Generally, red cultivars showed a higher concentration of phenolics and lactone content, highlighting two cultivars: ‘Carmesi’ and ‘Gaugin’. Microbiological fertilisers did not affect the content of the most abundant phenolic acids, or even lowered flavonoid content, which is in opposition to fertilisers’ contribution to overall lactone enhancement. Phenolic acids and flavonoids accumulated in the winter season, while lactones mounted up in autumn. In accordance, the more pleasant taste in all lettuce cultivars was evaluated in winter/spring season trials. Correlation coefficients showed a negative contribution of lactucopicrin, total lactone, caffeoylmalic acid, and chlorogenic acid concentration to lettuce overall taste.

Presented results showed that all three factors conjointly influenced all tested parameters, in a manner that cannot be predicted by testing a single one. Such a complex response needs further investigation regarding a possible enhancement of the fertiliser dosage in a continuous application and the selection of the cultivar regarding its interaction with environmental factors and agricultural practices. Our findings are important from the aspect of applied agriculture in which, in an ecological manner, it is possible to improve the nutrient status of lettuce by balancing the content of compounds that define consumers acceptance of different lettuce cultivars regarding a pleasant taste.

## Figures and Tables

**Table 1:** Main and interaction factor effects on the phenolic acids (mg/g DW) and total phenolic content (µg GA eq/g FW) in red and green lettuce.

Main Factors	3-CQA	5-CQA	CTA	CMA	2,3-DCTA	TPC
***Cultivar***						
Kiribati	1.87 ± 0.11 **a**	0.03 ± 0 **a**	4.36 ± 0.32 **b**	0.10 ± 0.01 **a**	5.70 ± 0.56 **a**	155.55 ± 15.24 **a**
Murai	3.29 ± 0.26 **bc**	1.51 ± 0.09 **cd**	3.34 ± 0.20 **a**	0.40 ± 0.04 **c**	17.10 ± 1.65 **c**	224.86 ± 16.43 **b**
Aquino	1.84 ± 0.09 **a**	1.20 ± 0.19 **bc**	4.06 ± 0.27 **b**	0.07 ± 0.004 **a**	6.07 ± 0.44 **a**	155.79 ± 9.40 **a**
Gaugin	3.84 ± 0.36 **c**	2.89 ± 0.31 **e**	3.58 ± 0.34 **a**	0.73 ± 0.06 **d**	24.89 ± 2.52 **d**	325.18 ± 30.64 **c**
Aleppo	2.93 ± 0.29 **b**	1.01 ± 0.26 **b**	4.35 ± 0.37 **b**	0.24 ± 0.01 **b**	10.45 ± 0.81 **b**	173.65 ± 20.24 **a**
Carmesi	5.11 ± 0.53 **d**	1.69 ± 0.21 **d**	3.35 ± 0.23 **a**	0.33 ± 0.02 **c**	23.49 ± 2.66 **d**	331.24 ± 25.66 **c**
***Fertiliser***						
Control	2.81 ± 0.16 **a**	1.33 ± 0.16	3.76 ± 0.26	0.24 ± 0.01 **a**	14.55 ± 1.77	222.00 ± 18.50 **ab**
EM Aktiv	3.73 ± 0.38 **b**	1.30 ± 0.22	3.95 ± 0.25	0.31 ± 0.03 **b**	14.79 ± 1.00	216.94 ± 14.02 **a**
Vital Tricho	3.18 ± 0.24 **a**	1.53 ± 0.22	3.88 ± 0.35	0.42 ± 0.03 **c**	14.63 ± 1.49	233.00 ± 19.12 **ab**
EM Aktiv + Vital Tricho	2.87 ± 0.31 **a**	1.39 ± 0.11	3.77 ± 0.29	0.27 ± 0.03 **ab**	14.49 ± 1.50	238.90 ± 26.77 **b**
***Growing season***						
Autumn	2.19 ± 0.17 **a**	0.77 ± 0.17 **a**	3.76 ± 0.33 **b**	0.59 ± 0.05 **c**	9.49 ± 0.86 **a**	283.49 ± 28.33 **c**
Winter	2.39 ± 0.18 **a**	1.60 ± 0.17 **b**	4.67 ± 0.38 **c**	0.31 ± 0.03 **b**	26.13 ± 2.94 **b**	229.25 ± 15.20 **b**
Spring	4.87 ± 0.47 **b**	1.80 ± 0.20 **c**	3.09 ± 0.15 **a**	0.03 ± 0 a	8.24 ± 0.53 **a**	170.39 ± 15.27 **a**
**Significance**						
Cultivar (C)	***	***	***	***	***	***
Fertiliser (F)	***	ns	ns	***	ns	**
Growing season (GS)	***	***	***	***	***	***
**Interaction factors**						
C × F	***	ns	*	***	***	***
C × GS	***	***	***	***	***	***
F × GS	***	**	*	***	***	***
C × F × GS	***	***	***	***	***	***

The data show the means (*n* = 3) ± SE. Values followed by the same letter are not significantly different at the 0.05% level of probability according to a Tukey’s test. Groups of the same factors with no letters are not different from each other. Asterisks indicate significant differences at * *p* ≤ 0.05; ** *p* ≤ 0.01; *** *p* ≤ 0.001; ns, non-significant. 3-CQA: chlorogenic acid, 5-CQA: neochlorogenic acid, CTA: caffeoyltartaric acid, CMA: caffeoylmalic acid, 2,3-DCTA: chicoric acid, and TPC: total phenolic content.

**Table 2:** Main and interaction factor effects on the flavonoid content (mg/g DW) in red and green lettuce.

Main Factors	Q-3MG-7G	Q-3G	Q-3Gc	Q-3MG	L-7G
***Cultivar***					
Kiribati	0.28 ± 0.04 **a**	0.76 ± 0.05 **b**	0.80 ± 0.09 **a**	0.69 ± 0.03 **a**	0.66 ± 0.01 **a**
Murai	0.55 ± 0.07 **b**	0.82 ± 0.06 **b**	0.86 ± 0.14 **a**	2.42 ± 0.15 **b**	4.20 ± 0.26 **c**
Aquino	0.30 ± 0.06 **a**	0.60 ± 0.02 **a**	0.80 ± 0.03 **a**	0.77 ± 0.02 **a**	0.63 ± 0.02 **a**
Gaugin	0.67 ± 0.04 **c**	1.05 ± 0.09 **c**	1.35 ± 0.26 **bc**	2.94 ± 0.26 **b**	6.96 ± 0.67 **e**
Aleppo	0.53 ± 0.05 **b**	1.00 ± 0.06 **c**	1.04 ± 0.11 **ab**	1.04 ± 0.08 **a**	1.92 ± 0.16 **b**
Carmesi	0.85 ± 0.06 **d**	1.68 ± 0.13 **d**	1.66 ± 0.25 **c**	6.37 ± 0.77 **c**	5.88 ± 0.59 **d**
***Fertiliser***					
Control	0.57 ± 0.06	1.06 ± 0.08 **b**	1.25 ± 0.20 **b**	2.60 ± 0.26 **b**	3.47 ± 0.35
EM Aktiv	0.55 ± 0.08	1.01 ± 0.06 **b**	1.12 ± 0.17 **ab**	2.56 ± 0.25 **b**	3.24 ± 0.23
Vital Tricho	0.51 ± 0.03	1.02 ± 0.08 **b**	1.05 ± 0.11 **ab**	2.32 ± 0.19 **ab**	3.45 ± 0.27
EM Aktiv + Vital Tricho	0.50 ± 0.05	0.83 ± 0.05 **a**	0.93 ± 0.11 **a**	2.02 ± 0.18 **a**	3.35 ± 0.34
***Growing season***					
Autumn	0.79 ± 0.05 **b**	1.13 ± 0.09 **b**	1.16 ± 0.13 **b**	1.53 ± 0.14 **b**	1.71 ± 0.15 **a**
Winter	0.77 ± 0.08 **b**	1.47 ± 0.10 **c**	1.61 ± 0.22 **c**	4.72 ± 0.46 **c**	6.22 ± 0.60 **c**
Spring	0.03 ± 0.03 **a**	0.35 ± 0.01 **a**	0.48 ± 0.09 **a**	0.86 ± 0.06 **a**	2.20 ± 0.13 **b**
**Significance**					
Cultivar (C)	***	***	***	***	***
Fertiliser (F)	ns	***	**	**	ns
Growing season (GS)	***	***	***	***	***
**Interaction factors**					
C × F	ns	***	ns	***	***
C × GS	***	***	***	***	***
F × GS	ns	***	ns	***	***
C × F × GS	*	***	***	***	***

The data show the means (*n* = 3) ± SE. Values followed by the same letter are not significantly different at the 0.05% level of probability according to a Tukey’s test. Groups of the same factors with no letters are not different from each other. Asterisks indicate significant differences at * *p* ≤ 0.05; ** *p* ≤ 0.01; *** *p* ≤ 0.001; ns, non-significant. Q-3MG-7G: quercetin-3-malonylglucoside-7-glucoside, Q-3G: quercetin-3-glucoside, Q-3Gc: quercetin-3′-O-glucuronide, Q-3MG: quercetin-3-O-(6″-O-malonyl)-glucoside, and L-7G: luteolin-7-glucoside.

**Table 3:** Main and interaction factor effects on the sesquiterpene lactone content (mg/g DW) and sensory attribute–overall taste in red and green lettuce.

Main Factors	Lactucopicrin	Dihydrolactucopicrin	Total Sesquiterpene Lactones	Sensory Attribute–Overall Taste
***Cultivar***				
Kiribati	0.15 ± 0.01 **bc**	0.003 ± 0 **a**	0.18 ± 0.01 **b**	2.93 ± 0.25
Murai	0.11 ± 0.01 **a**	0.006 ± 0.002 **ab**	0.12 ± 0.01 **a**	2.83 ± 0.23
Aquino	0.10 ± 0.01 **a**	0.014 ± 0.003 **c**	0.12 ± 0.02 **a**	3.08 ± 0.30
Gaugin	0.12 ± 0.02 **ab**	0.006 ± 0 **ab**	0.12 ± 0.02 **a**	2.87 ± 0.25
Aleppo	0.19 ± 0.03 **c**	0.005 ± 0.001 **ab**	0.21 ± 0.04 **b**	2.85 ± 0.21
Carmesi	0.37 ± 0.03 **d**	0.007 ± 0.001 **b**	0.39 ± 0.04 **c**	2.90 ± 0.21
***Fertiliser***				
Control	0.15 ± 0.02 **a**	0.003 ± 0 **a**	0.15 ± 0.02 **a**	2.87 ± 0.22
EM Aktiv	0.17 ± 0.01 **a**	0.008 ± 0.001 **b**	0.18 ± 0.02 **a**	2.85 ± 0.25
Vital Tricho	0.17 ± 0.01 **a**	0.009 ± 0.002 **b**	0.18 ± 0.02 **a**	3.03 ± 0.25
EM Aktiv + Vital Tricho	0.21 ± 0.03 **b**	0.007 ± 0.002 **b**	0.25 ± 0.04 **b**	2.89 ± 0.25
***Growing season***				
Autumn	0.24 ± 0.03 **c**	0.007 ± 0.002 **b**	0.26 ± 0.03 **c**	2.67 ± 0.24 **a**
Winter	0.12 ± 0.01 **a**	0.003 ± 0.001 **a**	0.13 ± 0.01 **a**	3.11 ± 0.22 **b**
Spring	0.16 ± 0.02 **b**	0.011 ± 0.002 **c**	0.18 ± 0.02 **b**	2.94 ± 0.26 **b**
**Significance**				
Cultivar (C)	***	***	***	ns
Fertiliser (F)	***	***	***	ns
Growing season (GS)	***	***	***	***
**Interaction factors**				
C × F	***	***	***	***
C × GS	***	***	***	***
F × GS	***	***	***	ns
C × F × GS	***	***	***	*

The data show the means (*n* = 3) ± SE for lactones and (*n* = 12) ± SE for the overall taste attribute. Values followed by the same letter are not significantly different at the 0.05% level of probability according to a Tukey’s test. Groups of the same factors with no letters are not different from each other. Asterisks indicate significant differences at * *p* ≤ 0.05; ** *p* ≤ 0.01; *** *p* ≤ 0.001; ns, non-significant.

**Table 4:** Correlation coefficients between tested parameters.

	TPC	DHLp	Lp	STL	Q-3G	Q-3MG	L-7G	3-CQA	CTA	CMA	2,3-DCTA	Q-3MG-7G	Q-3Gc	5-CQA	OT
**TPC**	1														
**DHLp**	−0.08	1													
**Lp**	0.27 *	0.26 **	1												
**STL**	0.24 **	0.22 **	0.96 **	1											
**Q-3G**	0.51 **	−0.32 **	0.12	0.07	1										
**Q-3MG**	0.57 **	−0.22 **	0.08	0.05	0.85 **	1									
**L-7G**	0.54 **	−0.24 **	−0.007	−0.04	0.66 **	0.82 **	1								
**3-CQA**	0.04	0.22 **	0.19 **	0.17 *	−0.26 **	−0.07	0.007	1							
**CTA**	0.09	−0.22 **	−0.15 **	−0.16 *	0.53 **	0.40 **	0.29 **	−0.30 **	1						
**CMA**	0.60 **	−0.05	0.05	−0.02	0.41 **	0.33 **	0.25 **	−0.05	0.23 **	1					
**2,3-DCTA**	0.55 **	−0.24 **	−0.01	−0.03	0.72 **	0.86 **	0.97 **	−0.06	0.41 **	0.29 **	1				
**Q-3MG-7G**	0.59 **	−0.34 **	0.17 *	0.13	0.79 **	0.68 **	0.61 **	−0.27 **	0.42 **	0.44 **	0.67 **	1			
**Q-3Gc**	0.49 **	−0.28 **	0.10	0.05	0.85 **	0.78 **	0.66 **	−0.21 **	0.51 **	0.34 **	0.71 **	0.73 **	1		
**5-CQA**	0.32 **	0.04	−0.06	−0.09	0.28 **	0.43 **	0.65 **	0.12	−0.05	0.10	0.56 **	0.15 *	0.33 **	1	
**OT**	−0.06	−0.005	−0.16 *	−0.16 *	0.07	0.12	0.16 *	−0.16 *	0.09	−0.14 *	0.15 *	−0.006	0.07	0.15 *	1

Asterisks indicate significant differences at * *p* ≤ 0.05; ** *p* ≤ 0.01; ns, non-significant. TPC: total phenolic content, DHLp: dihydrolactucopicrin, Lp: lactucopicrin, STL: total sesquiterpene lactone content, Q-3G: quercetin-3-glucoside, Q-3MG: quercetin-3-O-(6″-O-malonyl)-glucoside, L-7G: luteolin-7-glucoside, 3-CQA: chlorogenic acid, CTA: caffeoyltartaric acid, CMA: caffeoylmalic acid, 2,3-DCTA: chicoric acid, Q-3MG-7G: quercetin-3-malonylglucoside-7-glucoside, Q-3Gc: quercetin-3′-O-glucuronide, 5-CQA: neochlorogenic acid, and OT: overall taste.

**Table 5:** Growing and climatic data during three lettuce experiments.

	Autumn	Winter	Spring
	Growing data		
**Sowing**	19 September 2016	15 November 2016	5 April 2017
**Transplanting**	11 October 2016	27 December 2016	27 April 2017
**Harvest**	7 December 2016	5 April 2017	5 June 2017
**Vegetation period (days)**	58	100	40
	Climatic data		
**Average day temperature (°C)**	11.9	10.8	26.3
**Average night temperature (°C)**	5.7	1.8	15.3
**Average maximum temperature (°C)**	17.3	24.1	30.4
**Average minimum temperature (°C)**	−1.8	−6.9	12.8
**Average air humidity (%)**	87.2	81.5	74.2
**Photoperiod (h)**	11-9	9–13	14–15

## Data Availability

All new research data are presented in this contribution.

## References

[1] Todorović V., Rožić A., Marković S., Đurovka M., Vasić M. (2012). Influence of temperature on yield and earliness of lettuce grown in the winter period. Agro-Knowl. J..

[2] Tuladhar P., Sasidharan S., Saudagar P. (2021). Role of phenols and polyphenols in plant defense response to biotic and abiotic stresses. Biocontrol Agents and Secondary Metabolites.

[3] Adesso S., Pepe G., Sommella E., Manfra M., Scopa A., Sofo A., Tenore G.C., Russo M., Di Gaudio F., Autore G. (2016). Anti-inflammatory and antioxidant activity of polyphenolic extracts from *Lactuca sativa* (var. *Maravilla de Verano*) under different farming methods. J. Sci. Food. Agric..

[4] Manach C., Scalbert A., Morand C., Rémésy C., Jiménez L. (2004). Polyphenols: Food sources and bioavailability. Am. J. Clin. Nutr..

[5] Kim M.J., Moon Y., Tou J.C., Mou B., Waterland N.L. (2016). Nutritional value, bioactive compounds and health benefits of lettuce (*Lactuca sativa* L.). J. Food. Compos. Anal..

[6] Becker C., Kläring H.-P., Kroh L.W., Krumbein A. (2013). Temporary reduction of radiation does not permanently reduce flavonoid glycosides and phenolic acids in red lettuce. Plant Physiol. Biochem..

[7] Becker C., Klaering H.-P., Schreiner M., Kroh L.W., Krumbein A. (2014). Unlike Quercetin Glycosides, Cyanidin Glycoside in Red Leaf Lettuce Responds More Sensitively to Increasing Low Radiation Intensity before than after Head Formation Has Started. J. Agric. Food Chem..

[8] Cartea M.E., Francisco M., Soengas P., Velasco P. (2011). Phenolic Compounds in Brassica Vegetables. Molecules.

[9] Han Y., Zhao C., He X., Sheng Y., Ma T., Sun Z., Liu X., Liu C., Fan S., Xu W. (2018). Purple lettuce (*Lactuca sativa* L.) attenuates metabolic disorders in diet induced obesity. J. Funct. Food..

[10] Shulha O., Zidorn C. (2019). Sesquiterpene lactones and their precursors as chemosystematic markers in the tribe Cichorieae of the Asteraceae revisited: An update (2008–2017). Phytochemistry.

[11] Wesołowska A., Nikiforuk A., Michalska K., Kisiel W., Chojnacka-Wójcik E. (2006). Analgesic and sedative activities of lactucin and some lactucin-like guaianolides in mice. J. Ethnopharmacol..

[12] Chadwick M., Trewin H., Gawthrop F., Wagstaff C. (2013). Sesquiterpenoids lactones: Benefits to plants and people. Int. J. Mol. Sci..

[13] Moujir L., Callies O., Sousa P.M.C., Sharopov F., Seca A.M.L. (2020). Applications of Sesquiterpene Lactones: A Review of Some Potential Success Cases. Appl. Sci..

[14] Price K.R., Dupont M.S., Shepherd R., Chan H.W.S., Fenwick G.R. (1990). Relationship between the chemical and sensory properties of exotic salad crops—Coloured lettuce (*Lactuca sativa*) and chicory (*Cichorium intybus*). J. Sci. Food. Agric..

[15] Van Beek T.A., Maas P., King B.M., Leclercq E., Voragen A.G.J., De Groot A. (1990). Bitter sesquiterpene lactones from chicory roots. J. Agric. Food Chem..

[16] Seo M.W., Yang D.S., Kays S.J., Lee G.P., Park K.W. (2009). Sesquiterpene Lactones and Bitterness in Korean Leaf Lettuce Cultivars. HortScience.

[17] Hance P., Martin Y., Vasseur J., Hilbert J.-L., Trotin F. (2007). Quantification of chicory root bitterness by an ELISA for 11β,13-dihydrolactucin. Food Chem..

[18] Tamaki H., Robinson R.W., Anderson J.L., Stoewsand G.S. (1995). Sesquiterpene Lactones in Virus-Resistant Lettuce. J. Agric. Food Chem..

[19] Sung J.-S., Hur O.-S., Ryu K.-Y., Baek H.-J., Choi S., Kim S.-G., Luitel B., Ko H.-C., Gwak J.-G., Rhee J.-H. (2016). Variation in Phenotypic Characteristics and Contents of Sesquiterpene Lactones in Lettuce (*Lactuca sativa* L.) Germplasm. Korean J. Plant Resour..

[20] Mello J.C., Dietrich R., Meinert E.M., Teixeira E., Amante E.R. (2003). Efeito do cultivo orgânico e convencional sobre a vida-de-prateleira de alface americana (*Lactuca sativa* L.) minimamente processada. Food Sci. Technol..

[21] Menezes E.M.S., Fernandes É.C., Sabaa-Srur A.U.O. (2005). Folhas de alface lisa (*Lactuca sativa*) minimamente processadas armazenadas em atmosfera modificada: Análises físicas, químicas e físico-químicas. Food Sci. Technol..

[22] Di Monaco R., Miele N.A., Cabisidan E.K., Cavella S. (2018). Strategies to reduce sugars in food. Curr. Opin. Food Sci..

[23] Joshi H., Bishnoi S., Choudhary P., Mundra S. (2019). Role of Effective Microorganisms (EM) in Sustainable Agriculture. Int. J. Curr. Microbiol. Appl. Sci..

[24] Szczech M., Szafirowska A., Kowalczyk W., Szwejda-Grzybowska J., Włodarek A., Maciorowski R. (2016). The Effect of Plant Growth Promoting Bacteria on Transplants Growth and Lettuce Yield in Organic Production. J. Hort. Res..

[25] Babalola O.O. (2010). Beneficial bacteria of agricultural importance. Biotechnol. Lett..

[26] Liu X., Ardo S., Bunning M., Parry J., Zhou K., Stushnoff C., Stoniker F., Yu L., Kendall P. (2007). Total phenolic content and DPPH radical scavenging activity of lettuce (*Lactuca sativa* L.) grown in Colorado. LWT-Food Sci. Technol..

[27] Barickman T.C., Sublett W.L., Miles C., Crow D., Scheenstra E. (2018). Lettuce Biomass Accumulation and Phytonutrient Concentrations Are Influenced by Genotype, N Application Rate and Location. Horticulturae.

[28] Llorach R., Martínez-Sánchez A., Tomás-Barberán F.A., Gil M.I., Ferreres F. (2008). Characterisation of polyphenols and antioxidant properties of five lettuce varieties and escarole. Food Chem..

[29] Stojanović M., Maksimović V., Mutavdžić D., Petrović I., Jovanović Z., Savić S., Maksimović J.D. (2021). Determination of antioxidative and enzymatic activity in green and red lettuce cultivars affected by microbiological fertilisers and seasons. Emir. J. Food Agric..

[30] Fallovo C., Rouphael Y., Rea E., Battistelli A., Colla G. (2009). Nutrient solution concentration and growing season affect yield and quality of *Lactuca sativa* L. var. *acephala* in floating raft culture. J. Sci. Food. Agric..

[31] Oh M.-M., Carey E.E., Rajashekar C.B. (2009). Environmental stresses induce health-promoting phytochemicals in lettuce. Plant Physiol. Biochem..

[32] Avio L., Sbrana C., Giovannetti M., Frassinetti S. (2017). Arbuscular mycorrhizal fungi affect total phenolics content and antioxidant activity in leaves of oak leaf lettuce varieties. Sci. Hortic..

[33] Dudaš S., Šola I., Sladonja B., Erhatić R., Ban D., Poljuha D. (2016). The effect of biostimulant and fertilizer on “low input” lettuce production. Acta Bot. Croat..

[34] Kopta T., Pavlíková M., Sȩkara A., Pokluda R., Maršálek B. (2018). Effect of Bacterial-algal Biostimulant on the Yield and Internal Quality of Lettuce (*Lactuca sativa* L.) Produced for Spring and Summer Crop. Not. Bot. Horti. Agrobo..

[35] Stojanović M., Petrović I., Žuža M., Jovanović Z., Moravčević Đ., Cvijanović G., Savić S. (2020). The productivity and quality of *Lactuca sativa* as influenced by microbiological fertilisers and seasonal conditions. Zemdirbyste.

[36] Peters A.M., Haagsma N., van Amerongen A. (1997). A pilot study on the effects of cultivation conditions of chicory (*Cichorium intybus* L.) roots on the levels of sesquiterpene lactones in chicons. Z. Lebensm. Unters. Forsch. A.

[37] Lafarga T., Villaró S., Rivera A., Bobo G., Aguiló-Aguayo I. (2020). Bioaccessibility of polyphenols and antioxidant capacity of fresh or minimally processed modern or traditional lettuce (*Lactuca sativa* L.) varieties. J. Food Sci. Technol..

[38] Senizza B., Zhang L., Miras-Moreno B., Righetti L., Zengin G., Ak G., Bruni R., Lucini L., Sifola M.I., El-Nakhel C. (2020). The Strength of the Nutrient Solution Modulates the Functional Profile of Hydroponically Grown Lettuce in a Genotype-Dependent Manner. Foods.

[39] Sytar O., Zivcak M., Bruckova K., Brestic M., Hemmerich I., Rauh C., Simko I. (2018). Shift in accumulation of flavonoids and phenolic acids in lettuce attributable to changes in ultraviolet radiation and temperature. Sci. Hortic..

[40] Gan Y.Z., Azrina A. (2016). Antioxidant properties of selected varieties of lettuce (*Lactuca sativa* L.) commercially available in Malaysia. Int. Food Res. J..

[41] Assefa A.D., Choi S., Lee J.-E., Sung J.-S., Hur O.-S., Ro N.-Y., Lee H.-S., Jang S.-W., Rhee J.-H. (2019). Identification and quantification of selected metabolites in differently pigmented leaves of lettuce (*Lactuca sativa* L.) cultivars harvested at mature and bolting stages. BMC Chem..

[42] Rouphael Y., Kyriacou M., Vitaglione P., Giordano M., Pannico A., Colantuono A., De Pascale S. (2017). Genotypic variation in nutritional and antioxidant profile among iceberg lettuce cultivars. Acta Sci. Pol. Hortorum Cultus.

[43] Vidal V., Laurent S., Charles F., Sallanon H. (2019). Fine monitoring of major phenolic compounds in lettuce and escarole leaves during storage. J. Food Biochem..

[44] Nicolle C., Carnat A., Fraisse D., Lamaison J.L., Rock E., Michel H., Amouroux P., Remesy C. (2004). Characterisation and variation of antioxidant micronutrients in lettuce (*Lactuca sativa* folium). J. Sci. Food. Agric..

[45] Assefa A.D., Hur O.-S., Hahn B.-S., Kim B., Ro N.-Y., Rhee J.-H. (2021). Nutritional Metabolites of Red Pigmented Lettuce (*Lactuca sativa*) Germplasm and Correlations with Selected Phenotypic Characters. Foods.

[46] Ribas-Agustí A., Gratacós-Cubarsí M., Sárraga C., García-Regueiro J.-A., Castellari M. (2011). Analysis of Eleven Phenolic Compounds Including Novel p-Coumaroyl Derivatives in Lettuce (*Lactuca sativa* L.) by Ultra-high-performance Liquid Chromatography with Photodiode Array and Mass Spectrometry Detection. Phytochem. Anal..

[47] Giordano M., El-Nakhel C., Carillo P., Colla G., Graziani G., Di Mola I., Mori M., Kyriacou M.C., Rouphael Y., Soteriou G.A. (2022). Plant-Derived Biostimulants Differentially Modulate Primary and Secondary Metabolites and Improve the Yield Potential of Red and Green Lettuce Cultivars. Agronomy.

[48] Romani A., Pinelli P., Galardi C., Sani G., Cimato A., Heimler D. (2002). Polyphenols in greenhouse and open-air-grown lettuce. Food Chem..

[49] Selma M.V., Luna M.C., Martínez-Sánchez A., Tudela J.A., Beltrán D., Baixauli C., Gil M.I. (2012). Sensory quality, bioactive constituents and microbiological quality of green and red fresh-cut lettuces (*Lactuca sativa* L.) are influenced by soil and soilless agricultural production systems. Postharvest Biol. Technol..

[50] Ferreres F., Gil M.I., Castañer M., Tomás-Barberán F.A. (1997). Phenolic Metabolites in Red Pigmented Lettuce (*Lactuca sativa*). Changes with Minimal Processing and Cold Storage. J. Agric. Food Chem..

[51] Becker C., Urlić B., Jukić Špika M., Kläring H.-P., Krumbein A., Baldermann S., Goreta Ban S., Perica S., Schwarz D. (2015). Nitrogen Limited Red and Green Leaf Lettuce Accumulate Flavonoid Glycosides, Caffeic Acid Derivatives, and Sucrose while Losing Chlorophylls, Β-Carotene and Xanthophylls. PLoS ONE.

[52] Materska M., Olszówka K., Chilczuk B., Stochmal A., Pecio Ł., Pacholczyk-Sienicka B., Piacente S., Pizza C., Masullo M. (2019). Polyphenolic profiles in lettuce (*Lactuca sativa* L.) after CaCl_2_ treatment and cold storage. Eur. Food Res. Technol..

[53] El-Seedi H.R., El-Said A.M.A., Khalifa S.A.M., Göransson U., Bohlin L., Borg-Karlson A.-K., Verpoorte R. (2012). Biosynthesis, Natural Sources, Dietary Intake, Pharmacokinetic Properties, and Biological Activities of Hydroxycinnamic Acids. J. Agric. Food Chem..

[54] Park C.M., Jin K.-S., Lee Y.-W., Song Y.S. (2011). Luteolin and chicoric acid synergistically inhibited inflammatory responses via inactivation of PI3K-Akt pathway and impairment of NF-κB translocation in LPS stimulated RAW 264.7 cells. Eur. J. Pharmacol..

[55] Złotek U., Świeca M. (2016). Elicitation effect of Saccharomyces cerevisiae yeast extract on main health-promoting compounds and antioxidant and anti-inflammatory potential of butter lettuce (*Lactuca sativa* L.). J. Sci. Food. Agric..

[56] Baslam M., Garmendia I., Goicoechea N. (2011). Arbuscular Mycorrhizal Fungi (AMF) Improved Growth and Nutritional Quality of Greenhouse-Grown Lettuce. J. Agric. Food Chem..

[57] Geneva M.P., Stancheva I.V., Boychinova M.M., Mincheva N.H., Yonova P.A. (2010). Effects of foliar fertilization and arbuscular mycorrhizal colonization on *Salvia officinalis* L. growth, antioxidant capacity, and essential oil composition. J. Sci. Food. Agric..

[58] Bojilov D., Dagnon S., Kostadinov K., Filipov S. (2020). Polyphenol composition of lettuce cultivars affected by mineral and bio-organic fertilisation. Czech J. Food Sci..

[59] Ayuso-Calles M., García-Estévez I., Jiménez-Gómez A., Flores-Félix J.D., Escribano-Bailón M.T., Rivas R. (2020). Rhizobium laguerreae Improves Productivity and Phenolic Compound Content of Lettuce (*Lactuca sativa* L.) under Saline Stress Conditions. Foods.

[60] Marin A., Ferreres F., Barberá G.G., Gil M.I. (2015). Weather Variability Influences Color and Phenolic Content of Pigmented Baby Leaf Lettuces throughout the Season. J. Agric. Food Chem..

[61] Zhao X., Carey E.E., Young J.E., Wang W., Iwamoto T. (2007). Influences of Organic Fertilization, High Tunnel Environment, and Postharvest Storage on Phenolic Compounds in Lettuce. HortScience.

[62] Boo H.-O., Heo B.-G., Gorinstein S., Chon S.-U. (2011). Positive effects of temperature and growth conditions on enzymatic and antioxidant status in lettuce plants. Plant Sci..

[63] Sublett W., Barickman T.C., Sams C. (2018). The Effect of Environment and Nutrients on Hydroponic Lettuce Yield, Quality, and Phytonutrients. Horticulturae.

[64] Lee H., Oh I.-N., Kim J., Jung D., Cuong N.P., Kim Y., Lee J., Kwon O., Park S.U., Lim Y. (2018). Phenolic compound profiles and their seasonal variations in new red-phenotype head-forming Chinese cabbages. LWT.

[65] Galieni A., Di Mattia C., De Gregorio M., Speca S., Mastrocola D., Pisante M., Stagnari F. (2015). Effects of nutrient deficiency and abiotic environmental stresses on yield, phenolic compounds and antiradical activity in lettuce (*Lactuca sativa* L.). Sci. Hortic..

[66] Oh M.-M., Carey E.E., Rajashekar C.B. (2011). Antioxidant phytochemicals in lettuce grown in high tunnels and open field. Hortic. Environ. Biotechnol..

[67] Graziani G., Ferracane R., Sambo P., Santagata S., Nicoletto C., Fogliano V. (2015). Profiling chicory sesquiterpene lactones by high resolution mass spectrometry. Food Res. Int..

[68] Beharav A., Ben-David R., Malarz J., Stojakowska A., Michalska K., Doležalová I., Lebeda A., Kisiel W. (2010). Variation of sesquiterpene lactones in Lactuca aculeata natural populations from Israel, Jordan and Turkey. Biochem. Syst. Ecol..

[69] Bunning M., Kendall P., Stone M., Stonaker F., Stushnoff C. (2010). Effects of Seasonal Variation on Sensory Properties and Total Phenolic Content of 5 Lettuce Cultivars. J. Food Sci..

[70] Sessa R.A., Bennett M.H., Lewis M.J., Mansfield J.W., Beale M.H. (2000). Metabolite Profiling of Sesquiterpene Lactones from Lactuca Species. J. Biol. Chem..

[71] D’Antuono L.F., Ferioli F., Manco M.A. (2016). The impact of sesquiterpene lactones and phenolics on sensory attributes: An investigation of a curly endive and escarole germplasm collection. Food Chem..

[72] Drewnowski A., Gomez-Carneros C. (2001). Bitter Taste, Phytonutrients, and the Consumer: A Review. Am. J. Clin. Nutr..

[73] Gloess A.N., Schönbächler B., Klopprogge B., DAmbrosio L., Chatelain K., Bongartz A., Strittmatter A., Rast M., Yeretzian C. (2013). Comparison of nine common coffee extraction methods: Instrumental and sensory analysis. Eur. Food Res. Technol..

[74] Ruggieri F., Hance P., Gioia B., Biela A., Roussel P., Hilbert J.-L., Willand N. (2023). A Three-Step Process to Isolate Large Quantities of Bioactive Sesquiterpene Lactones from Cichorium intybus L. Roots and Semisynthesis of Chicory STLs Standards. Pharmaceuticals.

[75] Willeman H., Hance P., Fertin A., Voedts N., Duhal N., Goossens J.-F., Hilbert J.-L. (2014). A Method for the Simultaneous Determination of Chlorogenic Acid and Sesquiterpene Lactone Content in Industrial Chicory Root Foodstuffs. Sci. World J..

[76] Dragišić Maksimović J., Živanović B. (2012). Quantification of the Antioxidant Activity in Salt-Stressed Tissues. Methods Mol. Biol..

[77] (2012). General Guidelines for the Selection, Training and Monitoring of Selected Assessors and Expert Sensory Assessors.

[78] Ponce A., Roura S.I., Moreira M.d.R. (2011). Essential Oils as Biopreservatives: Different Methods for the Technological Application in Lettuce Leaves. J. Food Sci..

