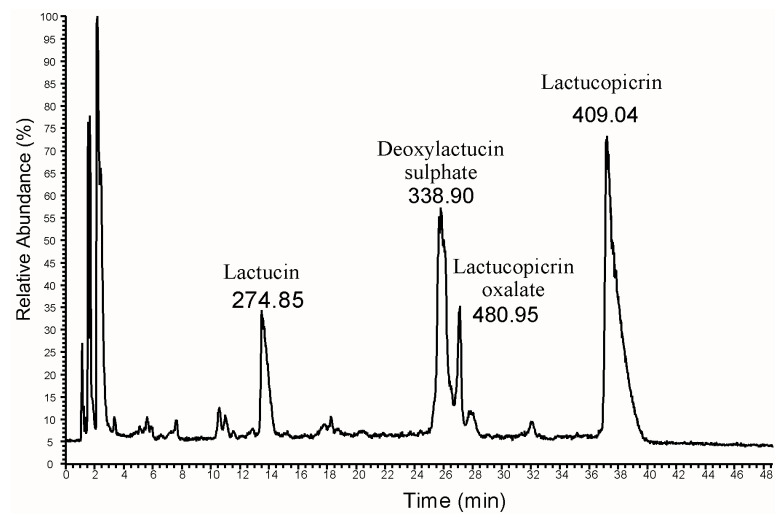# Correction: Stojanović et al. Phenolics and Sesquiterpene Lactones Profile of Red and Green Lettuce: Combined Effect of Cultivar, Microbiological Fertiliser, and Season. *Plants* 2023, *12*, 2616

**DOI:** 10.3390/plants15081142

**Published:** 2026-04-08

**Authors:** Milica Stojanović, Slađana Savić, Abigaël Delcourt, Jean-Louis Hilbert, Philippe Hance, Jelena Dragišić Maksimović, Vuk Maksimović

**Affiliations:** 1 Institute for Multidisciplinary Research, University of Belgrade, Kneza Višeslava 1, 11030 Belgrade, Serbia; draxy@imsi.bg.ac.rs (J.D.M.); maxivuk@imsi.bg.ac.rs (V.M.); 2 Institute for Vegetable Crops, Karađorđeva 71, 11420 Smederevska Palanka, Serbia; ssavic@institut-palanka.rs; 3 ICV-Institut Charles Viollette, UMRT 1158 BioEcoAgro, Univ. Lille, INRAE, Univ. Liège, Univ. Picardie Jules-Verne, YNCREA, Univ. Artois, Univ. Littoral Côte d’Opale, F-59000 Lille, France; abigael.delcourt@univ-lille.fr (A.D.); jean-louis.hilbert@univ-lille1.fr (J.-L.H.); philippe.hance@univ-lille.fr (P.H.); 4 Joint Laboratory University of Lille-Florimond-Desprez CHIC41Health, F-59655 Villeuneve d’Ascq, France

**Error in Figure**

In the original publication [1], there was a mistake in Figure 1 as published, where annotations for the last two peaks (Lactucopicrin and Lactucopicrin oxalate) have been marked inversely. The corrected version of Figure 1 appears below. The authors state that the scientific conclusions are unaffected. This correction was approved by the Academic Editor. The original publication has also been updated.